# Beneficial effects of PMX-DHP on vasopressor-resistant invasive meningococcal disease: a case report

**DOI:** 10.1097/MS9.0000000000005384

**Published:** 2026-07-14

**Authors:** Yuki Hattori, Nobutaka Chiba, Shingo Ihara, Jun Sato, Katsuhiro Nakagawa, Takeshi Saito, Kosaku Kinoshita

**Affiliations:** Division of Emergency and Critical Care Medicine, Department of Acute Medicine, Nihon University School of Medicine, Tokyo, Japan

**Keywords:** blood purification therapy, case report, circulatory insufficiency, cytokine, sepsis

## Abstract

**Introduction and importance:**

The efficacy of polymyxin B-immobilized fiber column direct hemoperfusion (PMX-DHP) for controlling inflammatory cytokines in patients with invasive meningococcal disease, which causes excessive release of inflammatory cytokines due to massive amounts of bacterial endotoxins, remains unclear.

**Case presentation:**

A 39-year-old male with invasive meningococcal disease, characterized by a blood endotoxin concentration ≥2000 pg/mL, developed circulatory failure unresponsive to vasopressors, respiratory failure, renal failure, and coagulation abnormalities. Three hours after admission, PMX-DHP was performed for 18 hours of treatment. Following the procedure, a reduction in the use of vasopressors became possible due to improved hemodynamics. Simultaneously, decreases in cytokine concentrations were observed.

**Clinical discussion:**

Although immunomodulatory therapy may improve the excessive inflammatory state in the early stages of sepsis, it may impair infection control and worsen outcomes during the later stages of immunodeficiency, making early initiation and prolonged use of PMX-DHP ideal.

**Conclusions:**

In a patient with invasive meningococcal disease complicated by septic shock, PMX-DHP was administered for a total of 18 hours, resulting in reductions in blood cytokine levels and dosages of vasopressors for rapidly progressing and vasopressor-resistant circulatory failure.

## Introduction

Endotoxin, the primary component of the outer membrane of gram-negative bacteria, activates immune cells and induces the release of cytokines, such as TNF-α, IL-1β, and IL-6, through signaling pathways centered on the NF-κB pathway^[^1^]^. Excessively released cytokines cause sustained inflammatory responses through the activation of vascular endothelial cells, increased vascular permeability, enhanced coagulation, and tissue edema. Consequently, this leads to organ dysfunction and, ultimately, to multiple organ failure^[^2^]^. Correlations have been demonstrated between the amount of cytokines released and sepsis complications and prognosis^[^3^]^. Therefore, early reduction in cytokine release is thought to improve the complications and prognosis of sepsis.

HIGHLIGHTS

- Invasive meningococcal disease can cause rapidly progressing circulatory failure due to excessive cytokine release triggered by elevated blood endotoxin levels.
- Immunomodulatory therapy, including PMX-DHP, may be attempted to suppress excessive cytokine responses during the initial treatment.
- Rapid implementation and prolonged use of PMX-DHP may improve circulatory failure by reducing cytokine levels.

Polymyxin B-immobilized fiber column direct hemoperfusion (PMX-DHP) (Toraymyxin^®^, Toray Medical Co., Ltd., Tokyo, Japan) has been reported to be effective as a treatment that suppresses excessive cytokine release by removing endotoxins from the blood^[^4^]^. Furthermore, reports indicate that performing PMX-DHP during infections caused by gram-negative bacteria, such as *Escherichia coli, Klebsiella pneumoniae*, and *Pseudomonas aeruginosa*, results in reduced cytokine levels^[^5,6^]^. Invasive infections by the meningococcus, a gram-negative diplococcus, are known to be frequently complicated by rapid circulatory failure, disseminated intravascular coagulation, Waterhouse–Friderichsen syndrome, and amputation due to limb necrosis because excessive cytokine release accompanies elevated blood endotoxin concentrations^[^7^]^. We report a case of invasive *Neisseria meningitidis* infection with an endotoxin concentration ≥2000 pg/mL (reference value ≤0.025 pg/mL) that progressed to septic shock and was treated with two consecutive sessions of PMX-DHP, totaling 18 hours. The results demonstrated not only a reduction in circulating cytokine levels but also a reduction in vasopressor requirements due to rapid improvement in circulatory failure. This case report has been prepared in line with the CARE Criteria^[^8^]^.

## Case presentation

The patient was a 39-year-old male who was transported to the emergency department because of generalized pain, a sore throat, difficulty moving, and a pallor. His initial vital signs included a temperature of 39.6 °C, a respiratory rate of 40 breaths per minute, a blood pressure of 63/47 mmHg, a heart rate of 132 beats per minute, and an oxygen saturation of 100% with a 6-L reservoir mask. His extremities were warm, he had erythema over his entire body, and he complained of widespread pain. After 2 L of Ringer’s acetate solution was administered, the patient’s blood pressure remained unresponsive, prompting the administration of norepinephrine (NAd) (0.45 μg/kg/min; γ), vasopressin (AVP) (5 U/h), and dexamethasone (0.6 mg/kg/day). Concurrently, ceftriaxone sodium (4 g/day) and clindamycin (2.7 g/day) were administered as antimicrobial agents. Blood tests revealed a white blood cell count of 10.8 × 10^3^/μL (reference range: 3.3–8.6 × 10^3^/μL), a platelet count of 82 × 10^3^/μL (reference range: 158–348 × 10^3^/μL), a C-reactive protein concentration of 8.86 mg/dL (reference range: ≤ 0.02 mg/dL), a blood urea nitrogen (BUN) concentration of 13.7 mg/dL (reference range: 8–20 mg/dL), a creatinine concentration of 3.79 mg/dL (reference range: 0.65–1.07 mg/dL), a D-dimer concentration of 35.97 µg/mL (reference range: ≤ 1 µg/mL), and a lactate concentration of 8.2 mmol/L (reference range: 0.5–1.6 mmol/L). He had no history of immunodeficiency, complement deficiency, or splenectomy. *N. meningitidis* was detected in his blood, sputum, and cerebrospinal fluid.

**Figure 1.**
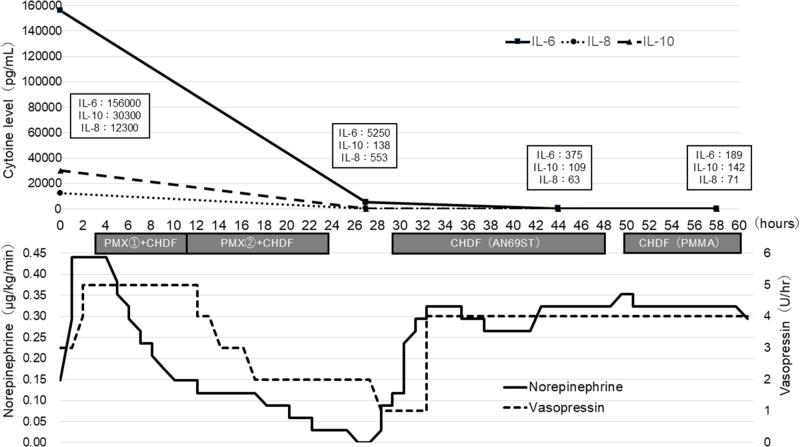
Trends in vasopressor dosages and cytokine levels.

PMX-DHP was performed twice consecutively for 9 hours per session, starting 3 hours after ICU admission. At the start of PMX-DHP, vasopressors were NAd 0.45 γ and AVP 5 U/h; by the end of the two 9-hour sessions, these concentrations were reduced to NAd 0.03 γ and AVP 2 U/h. The cytokine concentrations were 156 000 pg/mL for IL-6 (reference range: ≤ 4.0 pg/mL), 12 300 pg/mL for IL-8 (reference range: ≤ 2.0 pg/mL), and 30 300 pg/mL for IL-10 (reference range: ≤ 5.0 pg/mL) at the start of PMX-DHP. By the end of treatment, these concentrations decreased to 5250 pg/mL for IL-6, 553 pg/mL for IL-8, and 138 pg/mL for IL-10. Subsequently, no significant fluctuations in serum cytokine levels were observed (Fig. 1). FloTrac measurements of the hemodynamic parameters revealed a pre-initiation cardiac index (CI) of 4.9 L/min/m^2^ (reference range: 2.3–4.2 L/min/m^2^), and the systemic vascular resistance index (SVRI) was 765 dyne · sec/cm^5^ (reference range: 1800–2400 dyne · sec/cm^5^). At the end of the procedure, the CI was 3.4 L/min/m^2^, and the SVRI was 1477 dyne · sec/cm^5^; however, several hours later, the required dose of vasopressors increased (NAd 0.35 γ and AVP 4 U/h), with a CI of 5.2 L/min/m^2^ and an SVRI of 1020 dyne · sec/cm^5^. Vasopressor treatment was discontinued on Day 6. Renal replacement therapy was discontinued on Day 9. On the other hand, debridement was continued due to the development of dry black gangrene in both legs, ultimately resulting in amputation of both legs.

Effects of PMX-DHP on vasopressor dosages and cytokine levels: decreases in vasopressor dosages and cytokine levels were achieved. AN69ST is a polyethyleneimine-coated polyacrylonitrile membrane. PMMA is a polymethyl methacrylate membrane. PMX-DHP refers to polymyxin B-immobilized fiber direct hemoperfusion, and CHDF stands for continuous hemodiafiltration.

## Discussion and conclusion

Invasive meningococcal disease is known to rapidly become severe after onset and to have a high fatality rate of approximately 10–15%, even with appropriate treatment^[^7^]^. The cause is the massive release of bacterial endotoxins, triggering the excessive secretion of inflammatory cytokines, such as IL-6 and TNF-α, which induces a severe systemic inflammatory response known as a cytokine storm^[^9^]^. Treatment strategies involve controlling the causative infection and providing intensive care support. When such treatment proves difficult, immunomodulatory therapies, including low-dose hydrocortisone therapy to suppress excessive cytokine responses, the administration of IL-1 or IL-6 inhibitors, and blood purification therapies, such as PMX-DHP, are attempted^[^10,11^]^. This patient presented with respiratory failure, circulatory failure, coagulation abnormalities, and renal failure, suggesting the potential occurrence of a cytokine storm. PMX-DHP was performed for circulatory failure that was rapidly progressing despite the administration of vasopressors and low-dose hydrocortisone. This resulted in improved hemodynamics, allowing for the reduction of vasopressor dosages. Simultaneously, decreases in cytokine concentrations were observed.

Opinions are divided regarding the cytokine-reducing effect of PMX-DHP^[^12^]^. A large randomized controlled trial reported that the levels of major cytokines, such as IL-6, IL-1β, and IL-10, decreased over time in both the PMX-DHP and control groups, but no significant differences were observed between the groups. On the other hand, a cytokine-reducing effect was demonstrated in patients with septic shock due to intra-abdominal infection. After two PMX-DHP treatments, reductions in blood concentrations of IL-6, IL-10, and TNF-α were observed, along with decreased doses of norepinephrine and lactate levels, and improved SOFA scores^[^13^]^. Other reported effects of PMX-DHP include increased mean arterial pressure, improved cardiac output index and left ventricular work index, and a reduced need for renal replacement therapy^[^14^]^. These effects are also thought to be mediated by the reduction in cytokine levels^[^4^]^. Therefore, the improvement in hemodynamics observed in this case was also likely due to the reduction in cytokine levels. On the other hand, despite the observed reduction in the levels of cytokines, this patient experienced a recurrence of circulatory failure. A biphasic blood pressure reduction in the cytokine storm involves initial systemic vasodilation, increased vascular permeability, and a reduced circulating blood volume because of the rapid release of humoral factors such as inflammatory cytokines, complement activation products, and bradykinin^[^15^]^. The subsequent further decline is associated with increased NO production via inducible NO synthase, COX-2–induced prostaglandins, sustained cytokine release by macrophages, and immune dysregulation or impaired infection control due to anti-inflammatory responses^[^16^]^. In this case, no increase in cytokine levels was observed, and a recurrence of the reduced SVRI was noted. This suggests that vasodilation caused by prostaglandins and NO may have contributed to the increased demand for pressor agents. Therefore, even after controlling the initial response to circulatory failure, the need for hemodynamic monitoring and intervention against recurrence was considered.

In this case, these effects may have been observed because PMX-DHP was initiated just 3 hours after admission and administered continuously for 18 hours. While immunomodulatory therapy may improve the excessive inflammatory state in the early stages of sepsis, it may impair infection control and worsen outcomes during the later stages of immunodeficiency, making early initiation ideal^[^17^]^. Additionally, the standard duration for PMX-DHP has traditionally been 2 hours per session, with a second session performed 22–24 hours later^[^4^]^. However, a study that measured endotoxin concentrations at the inlet and outlet of the column after 24 hours of continuous PMX-DHP treatment of septic shock patients revealed that the endotoxin adsorption capacity of the column persisted even after 24 hours, with an average endotoxin removal rate of 74.4%. These results indicate that continuous endotoxin removal is achievable even with 24-hour continuous PMX-DHP^[^18^]^. Therefore, rapid implementation and prolonged use of PMX-DHP may be potentially beneficial for improving circulatory failure. Given that PMX-DHP was performed for 18 hours in this case, the effects of even longer durations remain unknown. Therefore, further investigations involving larger numbers of cases and including evaluation of the reproducibility of the effects are necessary.

## Data Availability

The datasets used and/or analyzed during the current study are available from the corresponding author upon reasonable request.
